# A Novel Method of Estimating Dose Responses for Polymer Gels Using Texture Analysis of Scanning Electron Microscopy Images

**DOI:** 10.1371/journal.pone.0067281

**Published:** 2013-07-02

**Authors:** Cheng-Ting Shih, Jui-Ting Hsu, Rou-Ping Han, Bor-Tsung Hsieh, Shu-Jun Chang, Jay Wu

**Affiliations:** 1 Department of Biomedical Engineering and Environmental Sciences, National Tsing Hua University, Hsinchu, Taiwan, R.O.C; 2 School of Dentistry, China Medical University, Taichung, Taiwan, R.O.C; 3 Department of Management Information Systems, Central Taiwan University of Science and Technology, Taichung, Taiwan, R.O.C; 4 Department of Medical Imaging and Radiological Science, Central Taiwan University of Science and Technology, Taichung, Taiwan, R.O.C; 5 Health Physics Division, Institute of Nuclear Energy Research, Taoyuan, Taiwan, R.O.C; 6 Department of Biomedical Imaging and Radiological Science, China Medical University, Taichung, Taiwan, R.O.C; Pennsylvania State University, United States of America

## Abstract

Polymer gels are regarded as a potential dosimeter for independent validation of absorbed doses in clinical radiotherapy. Several imaging modalities have been used to convert radiation-induced polymerization to absorbed doses from a macro-scale viewpoint. This study developed a novel dose conversion mechanism by texture analysis of scanning electron microscopy (SEM) images. The modified *N*-isopropyl-acrylamide (NIPAM) gels were prepared under normoxic conditions, and were administered radiation doses from 5 to 20 Gy. After freeze drying, the gel samples were sliced for SEM scanning with 50×, 500×, and 3500× magnifications. Four texture indices were calculated based on the gray level co-occurrence matrix (GLCM). The results showed that entropy and homogeneity were more suitable than contrast and energy as dose indices for higher linearity and sensitivity of the dose response curves. After parameter optimization, an *R* ^2^ value of 0.993 can be achieved for homogeneity using 500× magnified SEM images with 27 pixel offsets and no outlier exclusion. For dose verification, the percentage errors between the prescribed dose and the measured dose for 5, 10, 15, and 20 Gy were −7.60%, 5.80%, 2.53%, and −0.95%, respectively. We conclude that texture analysis can be applied to the SEM images of gel dosimeters to accurately convert micro-scale structural features to absorbed doses. The proposed method may extend the feasibility of applying gel dosimeters in the fields of diagnostic radiology and radiation protection.

## Introduction

Polymer gels were first proposed in 1993 as a radiation dose measurement tool to overcome the major limitation of Fricky gels; that is, the loss of spatial resolution caused by the ion diffusion effect [1]. Unlike traditional dosimeters, such as thermoluminescent dosimeters (TLD), ion chambers, and X-ray films, which can only measure one- or two-dimensional dose distribution in space, polymer gels can be shaped arbitrarily and have the benefit of dose measurement in the inherent three-dimensional nature. Therefore, polymer gels are promising as one of the available options for independent dose validation of modern radiotherapy techniques in clinical practice.

Pretreatment dose verification for radiotherapy treatment planning is an essential aspect of patient-specific quality assurance. Polymer gel dosimeters have been proposed to verify various photon delivery techniques, such as intensity-modulated radiation therapy (IMRT) [2], [3], RapidArc radiation therapy [4], stereotactic radiosurgery (SRS) [5], [6], and brachytherapy [7], [8]. In addition, the potential applications of polymer gels for the proton therapy [9], [10], boron-neutron capture therapy (BNCT) [11], [12], and targeted radionuclide therapy [13] are promising because of the tissue-equivalent characteristics regarding the effective atomic number, electron density, and stopping power ratio of gel to water. A dose conversion system for the irradiated gels should offer high accuracy and reliability for the demanding applications.

Monomers in an aqueous gel matrix are polymerized and crosslinked when gel dosimeters are irradiated. The degree of polymerization is proportional to the absorbed dose. Several imaging modalities have been used to determine the dose response in the macro scale based on the changes of physical or chemical properties of the irradiated gel. In magnetic resonance imaging (MRI), the spin-spin relaxation rate (*R*2) relies on the mobility of water molecules in the samples. Because the polymer-structure formation reduces the molecular mobility, MRI can be used to determine the level of polymerization through T2-weighted imaging to calculate the relative *R*2 map profile [1], [14], [15]. Based on the behavior that polymerization of co-monomers causes gel hardening, X-ray computed tomography (CT) [16], [17], [18] and optical computed tomography (OCT) [19], [20], [21] are used to estimate the changes in the attenuation of X-ray and visible light, respectively. OCT is usually considered as the gold standard of gel readouts due to its high sensitivity and repeatability. In addition, other modalities have been applied to gel measurements, including proton spectroscopy using the NMR spectrometer [22], [23], Raman spectroscopy [24], [25], and ultrasound tomography [26], [27].

In this study, we proposed a micro-scale dose conversion method using the morphological features of irradiated gels. Scanning electron microscopy (SEM), which allows measurements of surface topography, was used incorporated with quantitative image analysis. Four texture features, entropy, contrast, energy, and homogeneity, were extracted from the gray-level co-occurrence matrix (GLCM) [28] of the SEM images. The linearity and sensitivity of the texture index versus dose calibration curves were investigated. Through parameter optimization, we can evaluate the feasibility of using texture analysis on SEM images as a dose readout system for polymer gel dosimeters.

## Materials and Methods

### Preparation of Polymer Gels

The polymer gel used in this study was a derivative of the original NIPAM gel proposed by Senden et al. [29]. The weight percentage of the gelatin, the *N*-isopropyl-acrylamide (NIPAM) monomer, and the *N*,*N*′-methylene-bis-acrylamide (BIS) crosslinker was 5%, 5%, and 3%, respectively. A total of 5 mM tetrakis (hydroxymethyl) phosphonium chloride (THPC) was added as an oxygen scavenger. This recipe, henceforth referred to as n-NIPAM, was obtained from a preliminary study that optimized the gel composition using regular two-level fractional factorial designs [30]. The n-NIPAM gels were prepared under normoxic atmospheric conditions and poured into 12-mL glass vials (No. 9826, Pyrex, USA) which were subsequently wrapped with tinfoil to prevent ambient lighting and solidified at 4°C for 2 h before use. The n-NIPAM gel has been proven to achieve high linearity and sensitivity under a dose range of 0–20 Gy by CT readouts [31].

### Irradiation of Polymer Gels

The vial filled with n-NIPAM was placed horizontally at the center of a 15×15×4 cm^3^ PMMA phantom, which was subsequently sandwiched by 3-cm-thick solid water slabs for dose buildup. A medical linear accelerator (Clinac 21 iX, Varian, USA) with a gantry angle of 0° and a field size of 10×10 cm^2^ was used to irradiate the center of the vial with a source-to-axis distance (SAD) of 100 cm. Radiation doses of 5, 10, 15, and 20 Gy were delivered using a 6 MV photon beam at a dose rate of 400 MU/min. One vial of each batch was excluded from irradiation to serve as 0 Gy. After dose delivery, the vials were stored in a 4°C refrigerator for 48 h for polymerization to cease completely [31]. The gel vials were further moved to a −20°C liquid-nitrogen-cooled refrigerator for another 24 h of freeze drying before the gels were cut into 5-mm thin slices alone the long axis for SEM imaging.

### Texture Analysis of SEM Images

The microstructural features of the irradiated gels were examined using SEM (Jeol JSM-T330A, Peabody, USA). The gel slices were first mounted with a conductive adhesive and sputter-coated with gold palladium powder. 15-kV accelerated electron beams were then used to perpendicularly collide with the sample slice. The cross-sectional views of the sample surface were acquired at magnifications of 50×, 500× and 3500×.

The GLCM method [28], which extracts second-order statistical information, was applied to the 8-bit gray-level SEM images. The joint probability of two pixels with specific intensity levels away from a distance *d* was calculated along the specified direction *θ*. The spatial dependence matrix is defined as
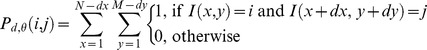
(1)where *I* is the input image, *N* and *M* are the height and width of *I*, respectively, *i* and *j* are the two specific intensity levels, respectively, and *d* denotes the offset between the two pixels (*x*, *y*) and (*x*+*dx*, *y*+*dy*). A normalized GLCM formula that can be treated as the probability is calculated as



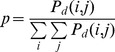
(2)The distance *d* was varied from 1 to 31 pixels, and the GLCM was averaged over four directions: 0°, 45°, 90°, and 135°.

Image texture analysis was performed on a slice-by-slice basis. The texture parameters, including entropy, contrast, energy, and homogeneity, were calculated from the normalized spatial dependence matrix to quantitatively represent the characteristics of the gel based on the likelihood that similar pixels are neighbors, as follows:
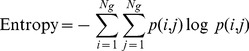
(3)

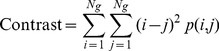
(4)

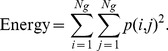
(5)

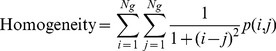
(6)where *N_g_* is the number of gray levels in the image. The details of the texture indices are described elsewhere [32]. In brief, entropy is used to measure the randomness of gray-level distribution, contrast represents the local variations presented in an image, energy reveals the regular patterns of pixels, and homogeneity represents the spatial similarity of image structures.

### Determination of Dose Response Curves

The SEM image of n-NIPAM with a matrix size of 1200×1600 was further divided into 16 sub-micrographs each with a size of 300×400. The texture indices were computed for each sub-image. Statistical outliers of the texture indices of sub-images were removed from the data to eliminate the effect of structural defects of the gel slice [33]. After outlier removal, the texture indices calculated from the remaining sub-images were averaged for the following process. The dose response curves (DRCs) for the four indices and the three magnifications were preliminarily determined using one gel sample for each dose level with distance *d = *10. The single-factor linear data regression model was applied. The linearity of the DRC is represented by the correlation of determination (*R* ^2^) of linear regression, and the sensitivity is defined as the absolute value of the slope of the DRC. After parametric optimization including the number of outlier exclusion *e* and the distance *d*, the optimized DRC was constructed using four SEM images acquired from four different sample tubes at each dose level.

### Dose Verification

Four sample tubes of the n-NIPAM polymer gels were delivered with absorbed doses of 5, 10, 15, and 20 Gy, respectively, using 6 MV photons at a dose rate of 400 MU/min. Three SEM images for each sample were acquired, and a total of 12 images for each dose level were used to calculate the mean homogeneity using the optimized scanning parameters and texture parameters. The prescribed dose and the measured dose were compared to evaluate the accuracy of dose conversion.

### Experimental Design and Statistical Analysis

The entire study can be divided into three parts: initial selection of the suitable texture indices and scanning parameters, optimization of the dose response curve, and validation of the dose conversion. Initially, one SEM image was acquired from a sample tube for each dose level and magnification level to investigate the appropriate magnification of SEM imaging and texture indices. The same set of the SEM images was also used to optimize the number of outlier exclusion *e* and the distance of offset *d* with the highest linearity. Secondly, for construction of the optimized dose response curve, four sample tubes, each acquired one SEM image, were used for each dose level. The standard error for each dose point was calculated. Finally, for the dose validation, another four sample tubes each acquired three SEM images at various locations were used for each prescribed dose to calculate the mean texture index and standard error, and subsequently converted to the measured dose.

## Results

**Figure 1:**
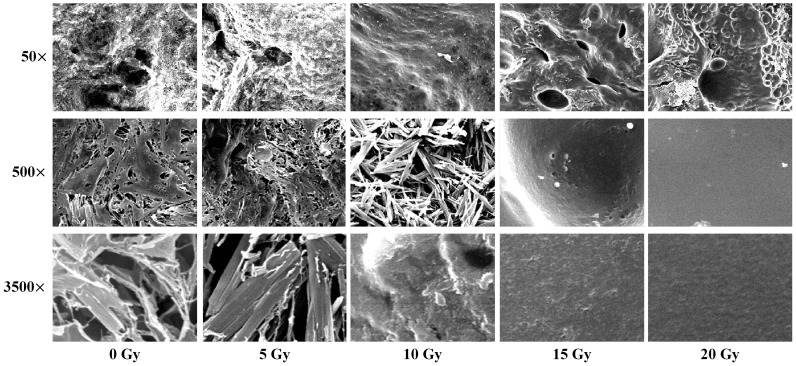
SEM images of the n-NIPAM gels with absorbed doses of 0, 5, 10, 15, and 20 Gy acquired at 50×, 500×, and 3500× magnifications. As the dose increased, the fibrous structures gradually disappeared at the 500× and 3500× micrographs.

Figure 1 shows the SEM images of the n-NIPAM gels received absorbed doses from 0 to 20 Gy. The spatial resolution for 50×, 500×, and 3500× magnifications was 1.26 µm, 125.94 nm, and 17.99 nm, respectively, and the field size was 3.05, 3.05×10^−2^, and 6.21×10^−4^ mm^2^. From a visual perspective, although sharp ridges and pits were easily recognized, the 50× micrographs did not reveal the detailed structure of gel fibers. The gray intensity in the local region changed rapidly due to the relatively large field of view (FOV). No regular patterns of roughness were observed as the absorbed dose increased. When the magnification increased to 500× and 3500×, the fiber-like structures with lengths of approximately 4.15 to 18.00 µm and diameters of 0.88 to 3.57 µm appeared. As the dose increased, the fiber exhibited a flake-like structure. After the dose was raised to 15 Gy, the strong polymerization caused the texture to disappear. Subsequently, the SEM image became smoother.

**Figure 2:**
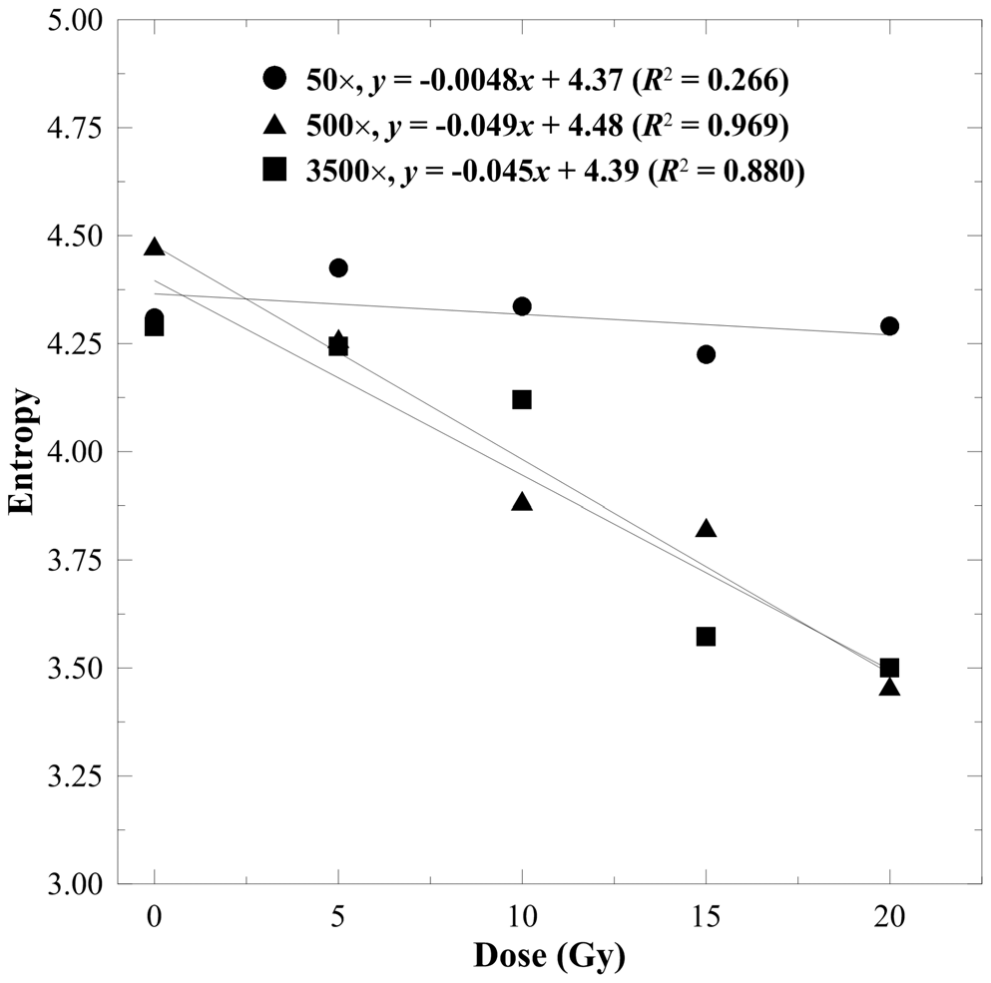
Entropy versus dose response curves for the n-NIPAM gels estimated from 50×, 500×, and 3500× magnified SEM images. A major downward trend was observed as the dose increased due to the increasing smoothness of the SEM micrographs.

**Figure 3:**
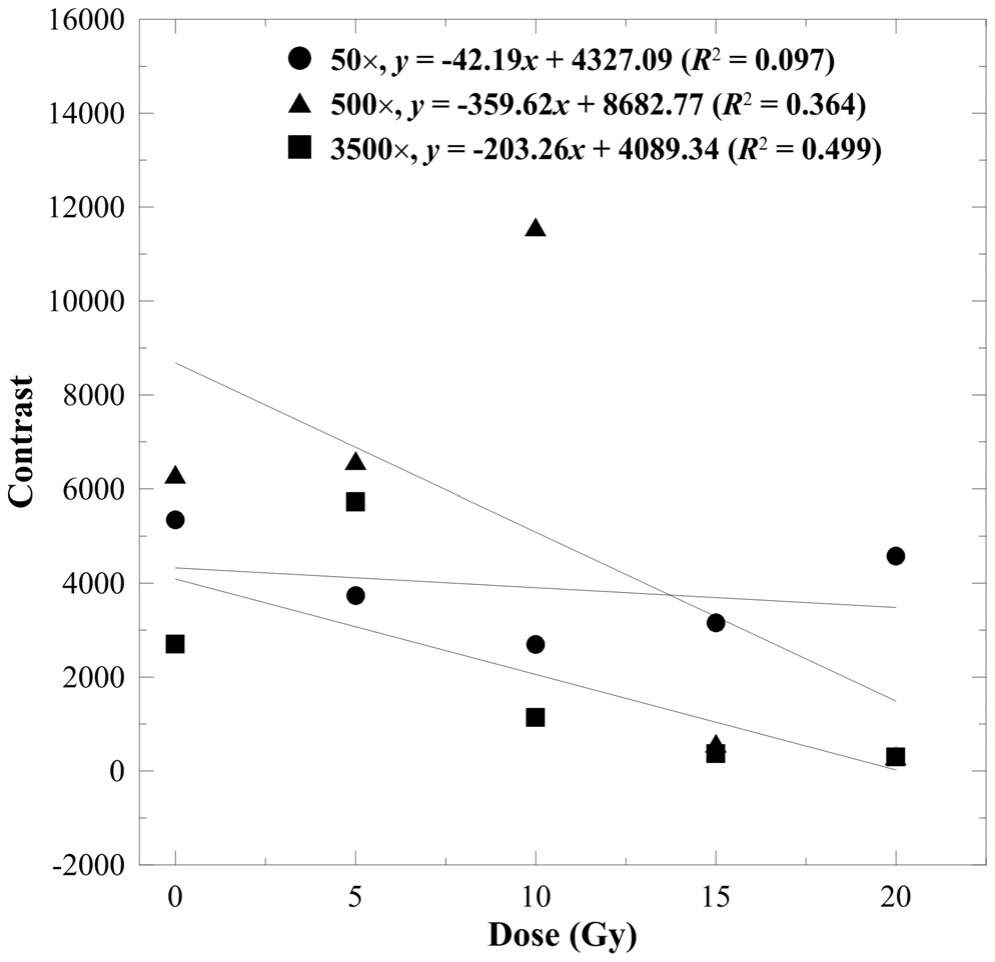
Contrast versus dose response curves for the n-NIPAM gels estimated from 50×, 500×, and 3500× magnified SEM images. A minor downward trend was observed as the dose increased at 500× and 3500× magnifications.

**Figure 4:**
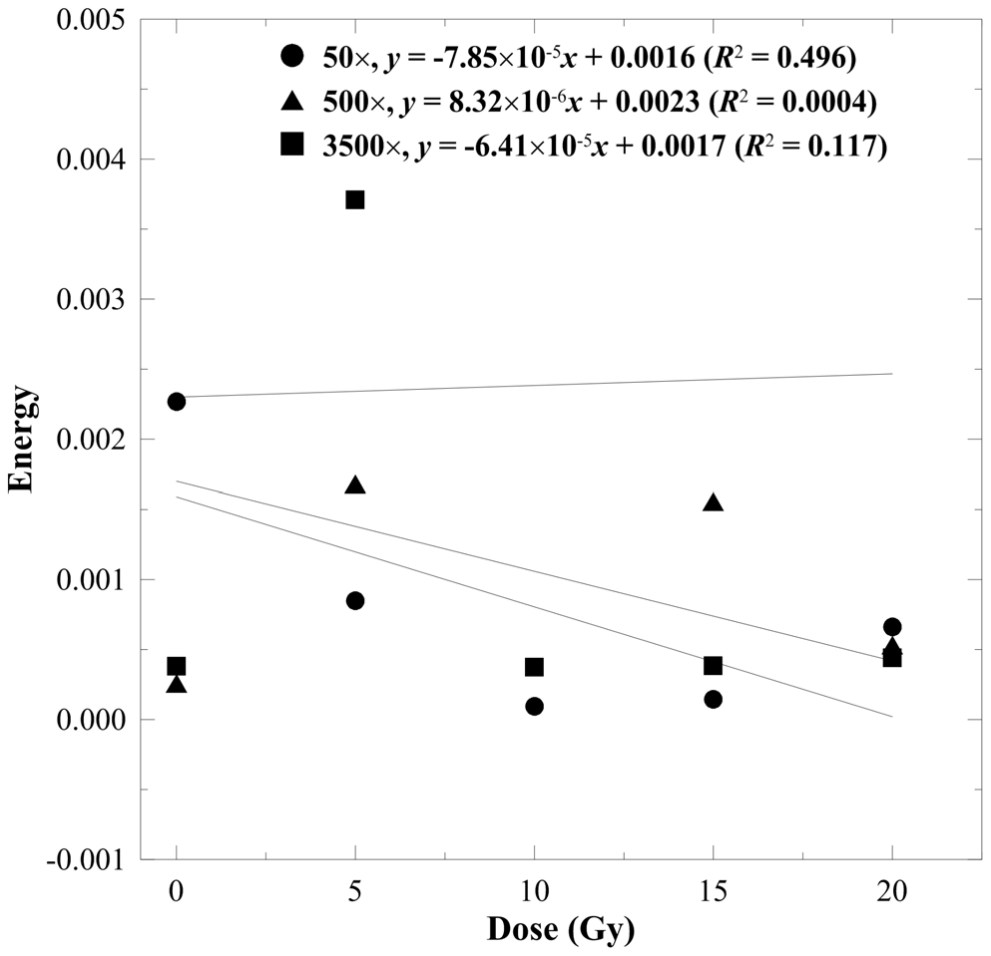
Energy-to-dose response curves for the n-NIPAM gels estimated from 50×, 500×, and 3500× magnified SEM images. Energy had a weak correlation to the absorbed dose for all magnifications.

**Figure 5:**
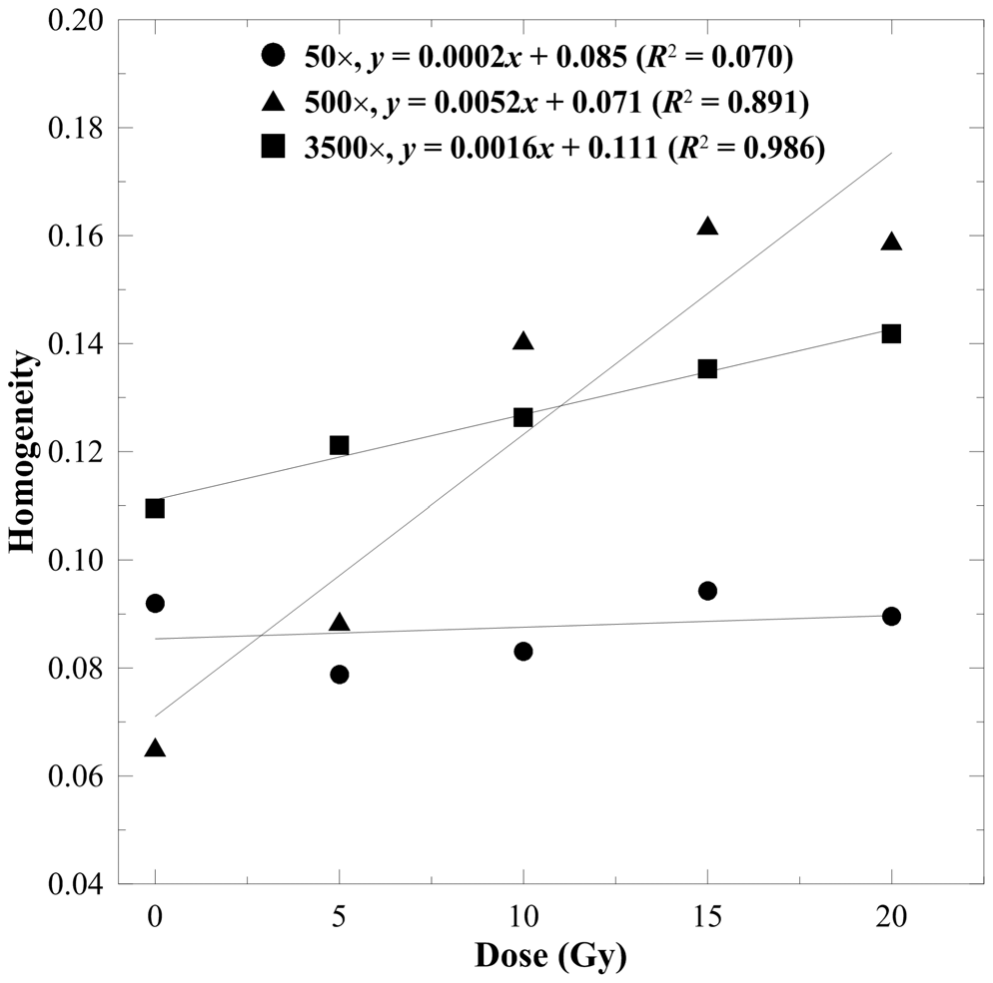
Homogeneity-to-dose response curves for the n-NIPAM gels estimated from 50×, 500×, and 3500× magnified SEM images. A linear relationship to the absorbed dose was observed. The *R* ^2^ values were 0.891 and 0.986 for 500× and 3500× magnifications, respectively.

Figures 2–5 display the texture index versus dose curves estimated from various magnifications of the SEM images with an offset of 10 pixels. The linearity of the 50× DRCs was fairly poor for all indices because the 50× micrographs cannot reveal substantial morphological variations under different absorbed doses. The DRCs of entropy and contrast are shown in Figure 2 and 3, respectively. These two indices exhibited a downward trend when the absorbed dose increased. This is mainly attributed to the increasing smoothness of the gel structures. The *R* ^2^ values of 500× and 3500× magnifications for the entropy were 0.969 and 0.880, respectively, indicating that entropy is a superior indicator to contrast which only achieved 0.364 and 0.499 for 500× and 3500× magnifications, respectively.

The energy-to-dose response curves are displayed in Figure 4, where the index had a weak correlation (*R* ^2^<0.5) to the absorbed dose for all magnifications. Figure 5 demonstrates that homogeneity had a positive correlation to the absorbed dose. The linearity was 0.891 and 0.986 for 500× and 3500× magnifications, respectively, indicating that homogeneity is suitable for dose conversion of the n-NIPAM gel. In addition, the slope of the 500× magnification was approximately triple that of the 3500×, implying that the morphological variations observed at 500× is more sensitive to the radiation dose. Entropy and homogeneity are superior to contrast and energy as dose indicators. Therefore, we further optimized these two texture indices at 500× and 3500× magnification levels.

**Figure 6:**
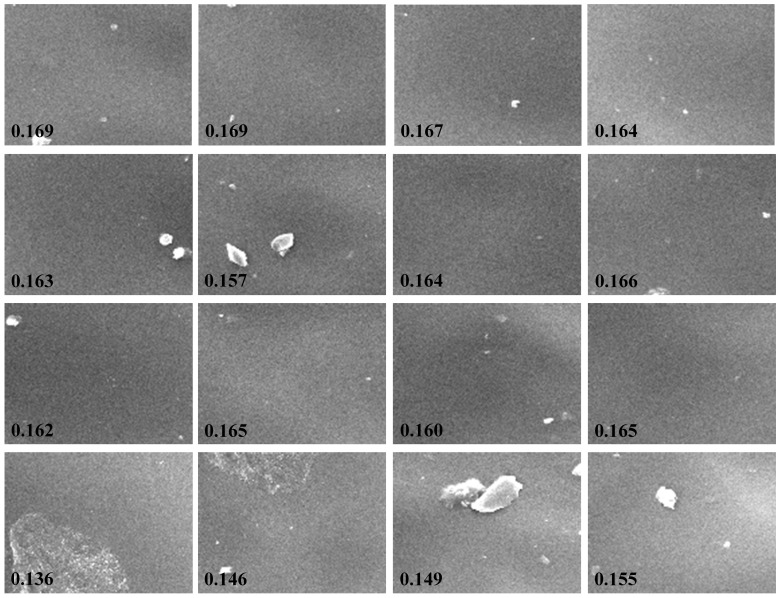
Sixteen sub-micrographs divided from the original 500× magnified SEM image irradiated with 20 Gy. The homogeneity is attached in the lower-left corner of each sub-image. Impurities in the gel g007matrix caused variations in the texture analysis results.

**Figure 7:**
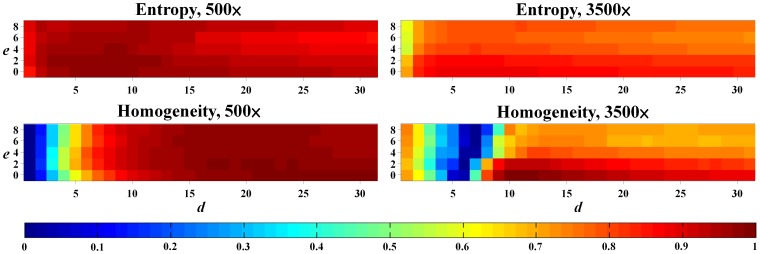
Linearity maps of entropy and homogeneity at 500× and 3500× magnifications. The *x* and *y* axes are the distance (*d*) and the exclusion number (*e*), respectively, and the pixel value in the matrix represents the *R* ^2^ value of the corresponding DRC. Homogeneity was more sensitive to *d* and *e* than entropy at both magnification levels.

Figure 6 shows the 16 sub-micrographs segmented from the original SEM image of n-NIPAM irradiated with 20 Gy at the 500× magnification. The homogeneity index of each sub-image is also shown at the lower left corner. The impurities in the gel matrix resulted in non-uniformity of the image and variations of texture analysis. The effects of excluding statistical outliers in the texture index calculation were further evaluated. The DRCs were constructed with different combinations of the exclusion number of statistical outliers *e* and the distance *d*, and their linearity and sensitivity were extracted. Figure 7 shows the linearity maps of entropy and homogeneity as a function of *e* and *d*. The pixel intensity represents the *R* ^2^ value of the corresponding DRC. For the 500× magnification, the exclusion number did not considerably affect the *R* ^2^ value. As the distance increased, the linearity of entropy remained nearly constant, whereas the linearity of homogeneity increased rapidly. The *R* ^2^ value stabilized after *d* >10. For the 3500× magnification, both texture indices decreased slightly when the exclusion number increased. For the distance, the linearity of homogeneity first decreased and subsequently increased and stabilized after *d* >12. By contrast, entropy had relatively consistent *R* ^2^ values, indicating that it is more robust than homogeneity as a dose indicator.

**Figure 8:**
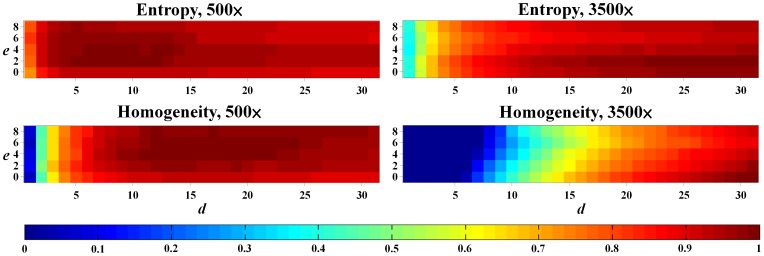
Sensitivity maps of entropy and homogeneity at 500× and 3500× magnifications. The *x* and *y* axes are the distance (*d*) and exclusion number (*e*), respectively, and the pixel value in the matrix represents the normalized absolute value of the slope of the corresponding DRC. Entropy had relatively stable results than homogeneity at both magnifications.

Figure 8 shows the sensitivity maps of entropy and homogeneity as a function of the exclusion number and the distance. The results of entropy were superior to those of homogeneity at a shorter distance and relatively stable throughout the entire variable domain. For the 500× magnification, the exclusion number had almost no impact on both indices. The average distance corresponding to 90% sensitivity was approximately 2.11 and 7.08 pixels for entropy and homogeneity, respectively. For the 3500× magnification, the sensitivity of homogeneity changed markedly when *d*<15. Higher pixel offsets of 12.77 and 28.41 were required to achieve 90% sensitivity for entropy and homogeneity, respectively.

**Figure 9:**
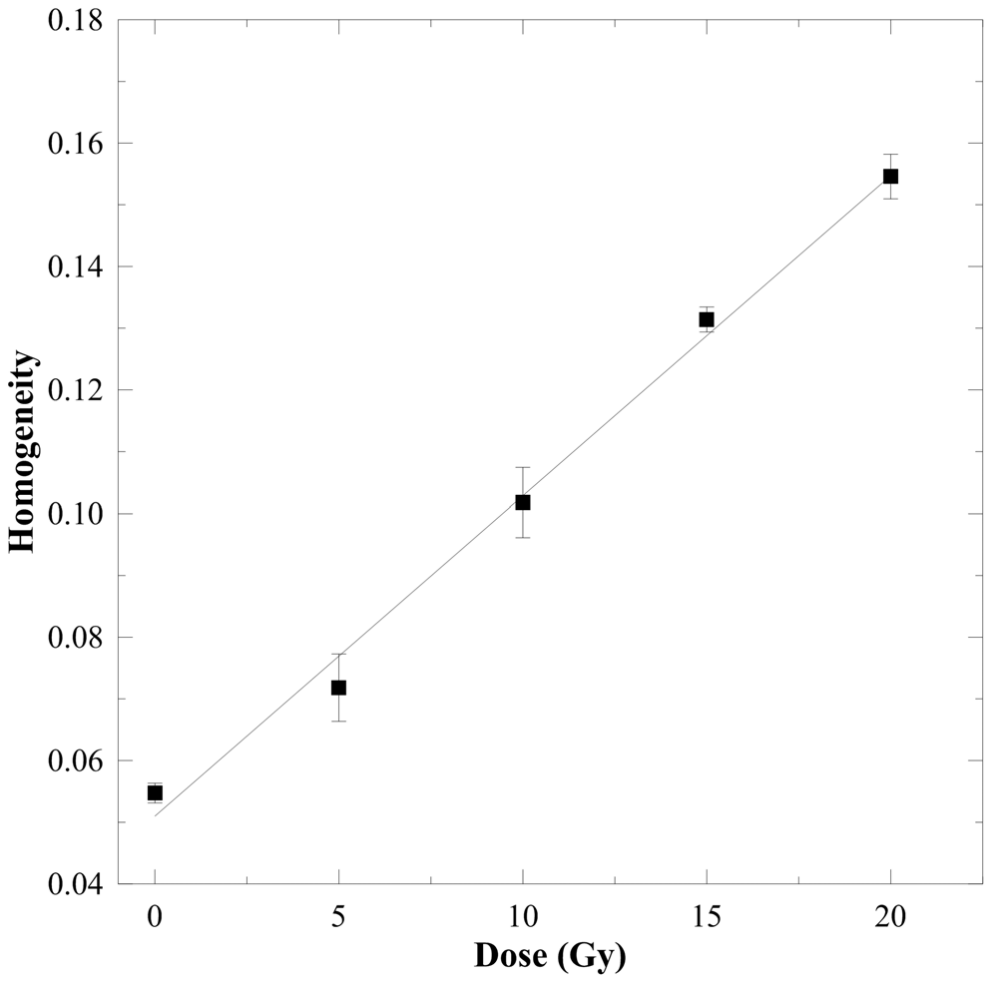
The optimized homogeneity-to-dose response curve for the n-NIPAM gels with 27 pixel offsets and no outlier exclusion at the 500× magnification. The linear fitting function is *y* = 0.0052*x*+0.051 (*R* ^2^ = 0.993), where *x* is the absorbed dose and *y* is the homogeneity.

Table 1 lists the optimized texture parameters based on linearity at 500× and 3500× magnifications. Although homogeneity at 3500× achieved the optimal *R* ^2^ value, its sensitivity was far lower than that of the other three combinations. Therefore, it was excluded from dose conversion. The homogeneity with 27 pixel offsets and no outlier exclusion at the 500× magnification was chosen as the optimized result. This parametric combination was further used for dose verification, and the corresponding DRC averaging over four samples of n-NIPAM gels at each dose level is shown in Figure 9. Table 2 compares the prescribed dose and the estimated dose using another 12 SEM images for each dose point with the dose response function of *y* = 0.0052*x*+0.051 (Figure 9), where *x* is the absorbed dose and *y* is the homogeneity. The percentage errors for 5, 10, 15, and 20 Gy were −7.60%, 5.80%, 2.53%, and −0.95%, respectively. The mean percentage error was −0.05%, indicating that the use of homogeneity on the 500× magnified SEM images is an accurate method for dose readouts of the n-NIPMA polymer gel dosimeters.

**Table 1:** Optimized texture parameters based on linearity at 500× and 3500× magnifications.

	500×				3500×			
Texture indices	*e*	*d*	Linearity (*R* ^2^)	Sensitivity	*e*	*d*	Linearity (*R* ^2^)	Sensitivity
Entropy	2	6	0.978	0.991	0	6	0.885	0.827
Homogeneity	0	27	0.993	0.903	0	11	0.995	0.477

Homogeneity at 3500× had relatively low sensitivity. Therefore, it was excluded from the dose calculation.

**Table 2:** Comparisons between the prescribed dose and the measured dose using the optimized scanning parameters and texture parameters.

Prescribed dose (Gy)	Mean homogeneity ± STD	Measured dose (Gy) ± STD	Percentage error
5	0.075±0.0067	4.62±0.811	−7.60%
10	0.106±0.0120	10.58±2.868	5.80%
15	0.131±0.0072	15.38±1.781	2.53%
20	0.154±0.0065	19.81±1.029	−0.95%
Mean percent error			−0.05%

## Discussion

After irradiation, NIPAM and BIS create a poly-NIPAM network in an aqueous matrix. BIS plays the role of a crosslinker to bind the C-C chain with the amide chain. The chemical reaction results in the changes in macro-scale characteristics such as the attenuation of visible light and the spin-spin relaxation rate (*R*2) of water molecules. This study proposes a novel dose readout mechanism based on SEM imaging and texture analysis. This micro-scale method can detect morphological changes caused by polymerizing and crosslinking of molecular structures at nanometer resolution. During the sample preparation, gel drying and cutting processes are required. Ice crystal formation due to a steep cooling rate and structural artifacts caused by cryofixation lead to embrittlement of gel structures. Irregular cross-sections and surface defects in the sample may appear after slicing and further increase the variation of texture index calculation. Additional impurities, residual gelatin particles, and aerosols mixed during gel manufacture may also affect texture analysis. Therefore, caution must be exercised during gel and slice preparation.

Texture analysis quantifies the heterogeneity of the irradiated gels by calculating the conditional probability of two adjacent pixels based on their gray levels, offsets, and orientations. We suggest that entropy and homogeneity are suitable for estimating the dose response of polymer gels. Entropy represents the randomness of gray-level distribution in the image. As the gel image becomes smoother, the entropy decreases monotonously. Homogeneity, on the other hand, indicates the surface roughness of the image structure. Lower homogeneity means larger variations between pixels due to the division of the square of gray level differences in Eq. 6. The homogeneity-to-dose response curve achieved the highest *R* ^2^ value after optimization of the magnifications, offsets, and exclusion numbers, indicating that it is the best index for dose conversion of the n-NIPMA gel. In addition, entropy is an alternative choice because of its stable linearity and sensitivity to the investigated parameters.

For various SEM magnifications, the 50× image with a pixel size of 1.26 µm is insufficient to reveal the detailed structure of the gel matrix. The wider FOV includes unwanted features, such as impurities and aerosols, which further affect the quality of texture analysis. For the 500× magnification, the spatial resolution rises to 125.94 nm, which is just suitable for observing the gel fibers. Consequently, the robust quantitative results can be achieved for the corresponding DRCs. For the 3500× magnification, the image resolution reaches 17.99 nm. The observed fibrous structure is too local and may have large fluctuations in gray intensities as the region of interest (ROI) changes, leading to less stable analysis results. In summary, at least a 500× magnification is required to fully demonstrate the morphological features of the n-NIPMA gel.

To reduce the fluctuations in the texture analysis results, the effect of excluding certain sub-images that contained impurities was examined. The results showed that although rejecting statistical outliers significantly reduced the standard deviation, it did not have notable influence on linearity and sensitivity. This occurs because the rejection of some sub-micrographs may also eliminate the edges of the structure and cause the mean value to be trapped at local optima that is away from the global mean. To increase the accuracy of dose conversion, the quality of polymer gels should be well controlled, and more micrographs could be scanning during DRC production to ensure that the global expectation values of the texture index are obtained.

Direction (*θ*) and distance (*d*) are the two essential parameters in GLCM calculation. The observation of the gel SEM images showed that n-NIPAM consisted of isotropic fibrous structures with a certain range of strip width and spacing (Figure 1). Therefore, four directions of the spatial dependence matrices were included and averaged, as they were treated in other study [34]. The selection of the distance depends on the fibrous structure of the gel and the applied texture index. Homogeneity is more sensitive to the distance than entropy and requires larger pixel offsets to achieve stable *R* ^2^ value. In addition, the magnification of SEM images, the type of monomers, and the ratio between each component of the gel also require different distance settings. Therefore, optimizing the distance is an essential step in performing the proposed readout method on gel dosimeters.

The BANG, PAG, and NIPAM polymer gels are primarily used for radiotherapy applications because the presented readout modalities cannot accurately quantify the macroscopic changes under an absorbed dose of less than 1 Gy. However, from a microscopic point of view, a small amount of latent polymerization has already occurred at the low dose range. SEM images with suitable texture indices can reflect the preliminary nature of polymerization. In the future, applications of polymer gel dosimetry and SEM readouts could be extended to quality assurance of radiation doses in diagnostic radiology and radiation protection. The influence of the dose rate dependency and the energy dependency on the proposed method needs to be further investigated. In addition to polymer gel dosimetry, the proposed SEM readout method may also be used to assess the degree of polymerization for hydrogels used in tissue engineering [35] and for industrial plastic products as bio-medical devices [36].

### Conclusion

In this study, we proposed a novel dose readout method that determined the dose response of the n-NIPMA gel using SEM images and GLCM-based texture analysis. The results showed that the use of 500× magnified images and homogeneity as the dose index with 27 pixel offsets can produce a DRC with optimum linearity and sensitivity. The average percentage error between the prescribed dose and the measured dose was −0.05%. In conclusion, the texture analysis can be applied to the SEM images of gel dosimeters to accurately convert the observed micro-scale structural features to absorbed doses. This method may extend the applications of gel dosimetry to the fields of diagnostic radiology and radiation protection.
